# Patterns of Crystallin Gene Expression in Differentiation State Specific Regions of the Embryonic Chicken Lens

**DOI:** 10.1167/iovs.63.4.8

**Published:** 2022-04-12

**Authors:** Zhiwei Ma, Daniel Chauss, Joshua Disatham, Xiaodong Jiao, Lisa Ann Brennan, A. Sue Menko, Marc Kantorow, J. Fielding Hejtmancik

**Affiliations:** 1 Ophthalmic Genetics and Visual Function Branch, National Eye Institute, National Institutes of Health, Bethesda, Maryland, United States; 2 Department of Biomedical Science, Charles E. Schmidt College of Medicine, Florida Atlantic University, Boca Raton, Florida, United States; 3 Department of Pathology, Anatomy and Cell Biology, Thomas Jefferson University, Philadelphia, Pennsylvania, United States

**Keywords:** RNA-sequencing, chicken, eye, lens, differentiation, crystallin

## Abstract

**Purpose:**

Transition from lens epithelial cells to lens fiber cell is accompanied by numerous changes in gene expression critical for lens transparency. We identify expression patterns of highly prevalent genes including ubiquitous and enzyme crystallins in the embryonic day 13 chicken lens.

**Methods:**

Embryonic day 13 chicken lenses were dissected into central epithelial cell (EC), equatorial epithelial cell (EQ), cortical fiber cell (FP), and nuclear fiber cell (FC) compartments. Total RNA was prepared, subjected to high-throughput unidirectional mRNA sequencing, analyzed, mapped to the chicken genome, and functionally grouped.

**Results:**

A total of 77,097 gene-specific transcripts covering 17,450 genes were expressed, of which 10,345 differed between two or more lens subregions. Ubiquitous crystallin gene expression increased from EC to EQ and was similar in FP and FC. Highly expressed crystallin genes fell into three coordinately expressed groups with *R*^2^ ≥ 0.93: CRYAA, CRYBB2, CRYAB, and CRYBA2; CRYBB1, CRYBA4, CRYGN, ASL1, and ASL; and CRYBB3 and CRYBA1. The highly expressed transcription factors YBX1, YBX3, PNRC1, and BASP1 were coordinately expressed with the second group of crystallins (*r*^2^ > 0.88).

**Conclusions:**

Although it is well known that lens crystallin gene expression changes during the epithelial to fiber cell transition, these data identify for the first time three distinct patterns of expression for specific subsets of crystallin genes, each highly correlated with expression of specific transcription factors. The results provide a quantitative basis for designing functional experiments pinpointing the mechanisms governing the landscape of crystallin expression during fiber cell differentiation to attain lens transparency.

Lens crystallins are most simply defined as proteins that are found in high concentration in the lens, fulfilling a structural role for transparency and refraction.^1^ They make up more than 90% of the water-soluble protein of the lens, resulting in the highest known protein concentration in any cell type. In the chicken, δ-crystallin constitutes 60% to 70% of the soluble protein of the embryonic lens, and the α- and β-crystallins make up most of the rest.^2^^,^^3^

In addition to the α and βγ-crystallin superfamily (called ubiquitous crystallins), which are found in all vertebrate lenses, there are also proteins termed “taxon-specific crystallins” that occur at high concentrations in the lens but are present only in selected species, although differential expression of even ubiquitous crystallins in different species and tissues has somewhat softened the lines between these groups. Many of the taxon-specific crystallins function as enzymes in non-lens tissues, where they are expressed at low concentrations. The simultaneous use of the same gene to encode a crystallin in the lens and an enzyme or other metabolic protein in non-lens tissues has given rise to the term “gene sharing.”^4^ Gene sharing may be followed by duplication and “subfunctionalization.”^5^^,^^6^ In view of their relationships with metabolic enzymes the taxon-specific crystallins are also called enzyme-crystallins. The δ-crystallin/arginosuccinate lyase (ASL) is a major crystallin in lenses of birds and reptiles and is the best studied enzyme-crystallin in the chicken.^7^ Although the protein product of the δ1-crystallin gene (ASL1) lacks enzymatic activity, δ2-crystallin (ASL) is an active enzyme. Other enzyme-crystallin genes include ε-crystallin/lactate dehydrogenase B,^8^^,^^9^ τ-crystallin/α-enolase (ENO1),^10^^,^^11^ ζ-crystallin/NADPH:quinone oxidoreductase (CRYZ),^11^ µ-crystallin/ornithine cyclodeaminase (CRYM),^12^ λ-crystallin/hydroxyacyl CoA dehydrogenase, and S-crystallins (GSTT1), which are inactive glutathione-S-transferases.^13^

Here we use high throughput RNA sequencing^14^^–^^16^ to identify the spectrum and magnitude of both ubiquitous and enzyme crystallin gene expression changes occurring during the transition from lens central epithelial cells to equatorial epithelial cells to cortical fiber cells to central nuclear fiber cells in the embryonic day 13 chicken lens.

## Methods

### Embryonic Lens Microdissection

**Figure 1.**
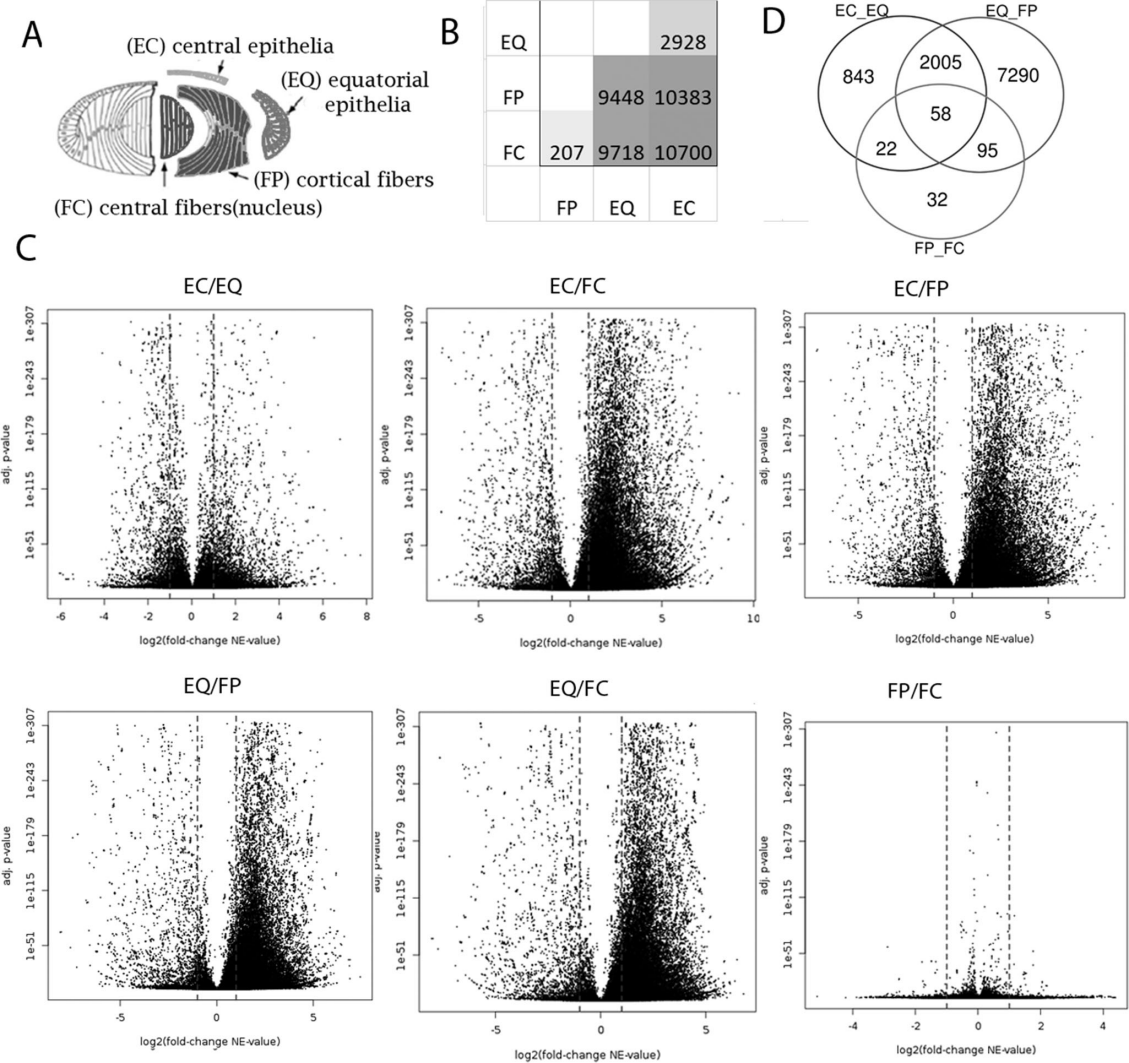
Identification of differentially expressed transcripts. (**A**) Lens zones from which RNA was isolated: EC, EQ, FP, and FC. (**B**) Number of nonunique differentially expressed transcripts between each embryonic lens region in pairwise comparison (**C**) Volcano plots showing differentially expressed gene-specific transcripts between lens regions. (**D**) Venn diagram of transcripts differentially expressed between developmentally sequential zones of the lens.

Fertilized chicken eggs (B&E Eggs, York Springs, PA, USA) were incubated to embryonic day 13 at 99.8°F, in a humidified incubator with automated rotation (GQF Manufacturing Company Inc., Savannah, GA, USA). Differentiation-state analysis of embryonic chicken lenses was performed after microdissection of 100 embryonic day 13 (E13) chicken lenses into four distinct zones (Fig. 1A) that represent a continuum of lens cell differentiation states: lens central epithelium (EC), equatorial epithelium (EQ), cortical fibers (FP), and central fibers (FC) as described previously by Walker and Menko.^17^

### RNA Sequencing of Pooled Microdissected Chicken Lenses

RNA sequencing was as described previously, using the raw sequencing results from Chauss et al.^18^ and deposited into the GEO database ascension number GSE53976. Briefly, two independent sets of 100 chicken lenses were microdissected and pooled and total RNA was prepared for each sample by established protocols (Trizol; Invitrogen, Carlsbad, CA, USA). Total RNA was analyzed for quality and subjected to mRNA directional sequencing library preparation (Illumina, San Diego, CA, USA) and analyzed for quality using the Agilent Technologies 2100 Expert Bioanalyzer (Santa Clara, CA, USA). Prepared libraries were then sequenced unidirectionally with the Genome Analyzer IIx. Mappable reads were mapped to the chicken genome (Galgal6; NCBI) using Partek Flow (https://partekflow.cit.nih.gov/flow) on the NIH Biowulf supercomputing cluster and then merged and analyzed by the GenoMatix genome analyzer (Genomatix Software Inc, Ann Arbor, MI, USA). A total of 77,097 gene-specific transcripts covering 17,450 genes were identified. Approximately 8 to 13 million reads were uniquely mapped to the chicken genome per microdissected lens area sample. A total of 10,345 genes exhibiting significantly different (adjusted *P* value < 0.05) expression levels between lens sub-regions were identified (Figs. 1B–D).

The reads in the input data set were analyzed, and for each transcript a normalized expression value (NE) was calculated from the read distribution as implemented in the Genomatix Suite (Genomatix Software Inc, MI).^19^ The NE value is based on the number of reads located in the exons of the transcript and is normalized to the length of the transcript and the density of the data set. For the differential expression analysis, a comparison of the expression values of the two input data sets was made on a transcript level by the Audic/Claverie method and then on a gene level. A total of 10,345 gene-specific transcript differences showed a mean log2 fold change of expression including 843 between EC and EQ only, 2005 between EC and EQ and between EQ and FP, 58 between all adjacent compartments, 22 between EC and EQ and between FP and FC, 7290 between EQ and FP only, 95 between EQ and FP and between FP and FC, and 32 between FP and FC only (Figs. 1B, 1D). Examination of the two independent sets of samples from each lens region using PCA show relatively good agreement of each (Supplementary Fig. S1). The two epithelial and fiber samples are well separated by PC1, whereas the central and equatorial epithelia are well separated on PC2. The fineness of this plot and the lack of discrimination between the central and peripheral fiber samples is consistent with their similarity in expression profiles, with only 207 differentially expressed genes (Fig. 1D). For differential methylation studies bisulfite sequencing was performed by Novogene (Sacramento, CA, USA) using an Illumina HiSeqTM2500/MiSeq platform using their standard protocols followed by CASAVA base calling and Trimmomatic read trimming and alignment to the galgal6 reference genome using Bismark.^20^ These are described more fully in Chauss et al. (submitted).

### Statistical Analysis

Further analysis focused on expression of all crystallin and other highly expressed genes. A group of house-keeping genes (GAPDH, ACTB, HMBS, H6PD, RPL4, RPLP0, RPLP1, TFRC, ALB, B2M, SDHA, TBP, TUBB and YWHAZ) was selected based on previous successful use as RNASeq controls,^21^^,^^22^ and genes were normalized to the average of their NE-values normalized to expression in the EC (Supplementary Fig. S2). The coefficient of determination of expression levels of various genes as indicated by their NE across the four lens regions was calculated using the numeric regression function included in the Golden Helix Sequence Variation Suite. Venn diagrams were prepared using the Venny 2.1 online program.^23^ Transcription factor binding sites were predicted using the Genomatix Software Suite (described online at: Transcription factor binding sites represented by Genomatix weight matrices), which returns a matrix representing the DNA binding site and the transcription factor or factors predicted to bind to that matrix.

## Results

### Highly Expressed Genes in Microdissected Lens Sub-Regions

Overall, the ubiquitous crystallins, along with ASL1 (CRYD1, δ1-crystallin), were the most highly expressed genes in all compartments of the lens, comprising 47% of transcripts in the central epithelia and increasing to 89% in the FP and FC regions (Table 1). The ubiquitous crystallins (with NEs averaging 12–362) and especially the taxon-specific crystallins (NEs averaging 34 – 2,155) are expressed at significantly higher levels than any of the other groups of highly expressed genes including protein synthesis and transcription factors (NEs:7–150), intermediate filaments, cytoskeleton, intercellular junctions, or ECM (NEs: 11–36), and metabolic enzymes involved in glycolysis and intermediary metabolism or and protease or inflammatory inhibitors (NEs: 12–65).

**Table 1.** Top 30 Highly Expressed Genes in the 4 Compartments of the 13 dpf Chick Lens Gene Symbols Are Color Coded With *pink* = Alpha-Crystallins, *blue* = Beta-Crystallins, *green* = Taxon-Specific Crystallins, *purple* = Anti-Inflammatory Factors, *lavender* = metaBolic Enzymes, *blue green* = Cytoskeleton and Junctional Proteins, tan = Protein Synthesis (*light*) and Transcription or Regulatory (*dark*) Factors. Loci of Unknown Function Are Shown in *dark green*


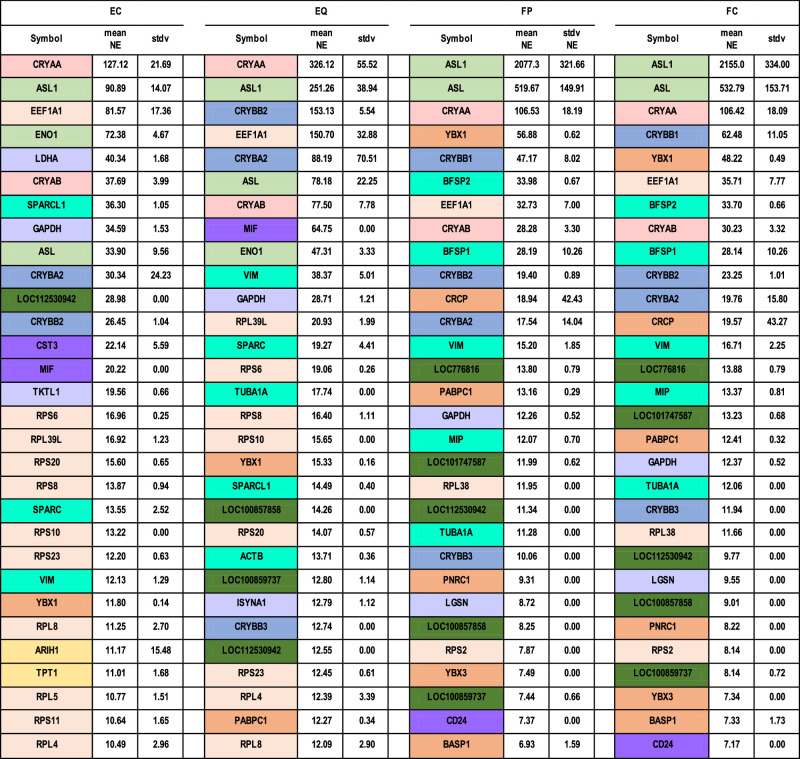

### Comparison of Ubiquitous Crystallin Gene Expression in Microdissected Lens Sub-Regions

**Figure 2.**
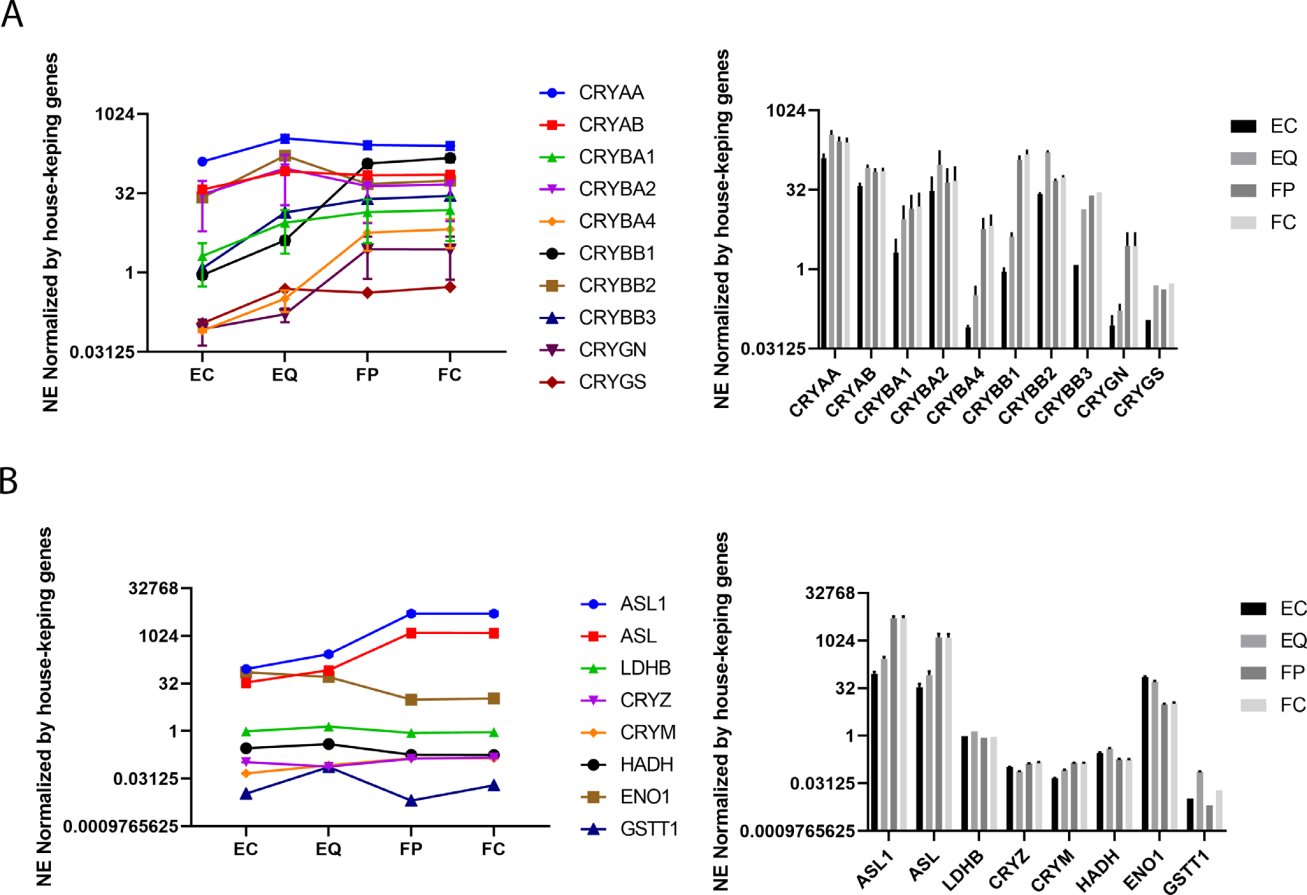
Crystallin expression in lens compartments. (**A**) Expression levels of ubiquitous crystallins. Initial measurements in NE were normalized for changing levels of highly expressed proteins by dividing by the averaged expression level of a standard set of housekeeping genes in each lens compartment. Expression levels are shown as a line graph in the left panels and bar graph with standard deviations in the right panels. ASL1 levels are included in panel **B**. NE levels are shown on a logarithmic scale to accommodate the wide variation in expression. (**B**) Expression levels of taxon specific crystallins. Initial measurements in NE shown in the *top panels* were normalized as above. Expression levels are shown as a *line graph* in the *left panels* and *bar graph* with 2 standard deviation limits shown in the *right panels*. NE levels are shown on a logarithmic scale to accommodate the wide variation in expression.

All ubiquitous crystallins were expressed in each lens compartment at levels ranging from an NE of 0.06 (for CRYGN in the EC compartment, not included in the top 30 transcripts shown in Table 1) to 326 for CRYAA in the EQ compartment). CRYAA, CRYAB, CRYBA2 and CRYBB2 are highly expressed in the central epithelia, with CRYBB3 joining in the EQ and CRYBB1 also joining in the FP. All crystallin genes were more highly expressed in the EQ compared with EC, and no crystallin gene expression exhibited a significant difference between the FP and FC compartments (Fig. 2A). Because estimates of ubiquitous crystallin mRNA prevalence might be skewed by the large amounts of ASL1 mRNA synthesized during differentiation from equatorial epithelia to fiber cells (Fig. 2B), their expression was normalized relative to a panel of 14 genes commonly used as controls for expression in the literature (see Methods, Supplementary Fig. S2). Although the control genes showed some variability of expression, when expression of the ubiquitous crystallins was normalized by the average expression of the control group (so that a corrected NE of 1 is the average of all the control group expression levels in that lens compartment), except for CRYBB1, CRYBA4, and CRYGN as previously mentioned, ubiquitous crystallin expression as a fraction of all mRNAs is seen to be essentially level among the equatorial epithelia, peripheral fiber, and central fiber compartments (Fig. 2A), suggesting that the apparent decrease in expression of the remaining genes from the epithelia to the fiber compartments was indeed due to extremely high levels of expression of ASL1.

The mRNA levels of the ubiquitous crystallins are seen to fall into three main groups by pattern of expression: group 1, including CRYAA, CRYBB2, CRYAB, and CRYBA2 (with CRYGS more loosely), in which mRNA levels increase from the central to equatorial epithelia and then stay constant or fall off slightly in the fibers; group 2, including CRYBB1, CRYBA4, CRYGN, ASL1 (CRYD1, a taxon-specific crystallin, but included here because it is the predominant crystallin in the chick lens) with ASL (ASL, CRYD2), in which mRNA levels increase dramatically from the epithelia to the fiber cells; and finally group 3 comprising CRYBB3 and CRYBA1, which are also loosely correlated with GSTT1. Each of these three main groups shows correlations greater than 0.93 (Table 2) but no significant correlation with crystallins in the other groups. CRYGS is not correlated strongly with either group but shows some suggestive correlation with group 1 that does not reach statistical significance.

**Table 2.** Coefficients of Determination of Ubiquitous and Taxon-Specific Crystallins. Highly Expressed Crystallins Are Shown at the Top of the Table. Correlation Coefficients Are Color Coded With *red* > 0.95, *pink* > 0.9, and *blue* < 0.5. *R*^2^ Values > 0.902505436426379 Have *P* < 0.05. Crystallins Are Shaded by Category: *dark pink*: Ubiquitous; *light pink*: Taxon-Specific for Birds and Reptiles; *blue*: Taxon-Specific for Mammals; *green*: Taxon-Specific for Mollusks and Cephalopods. Crystallin Genes With CpG Groups More Highly Methylated in Epithelia. *Crystallin Genes With CpG Groups More Highly Methylated in Fibers


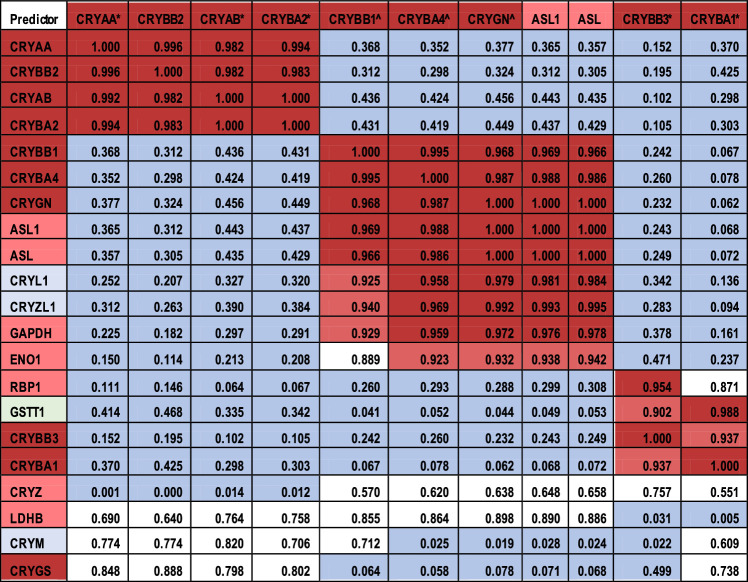

### Comparison of Taxon-Specific Crystallin Gene Expression in Microdissected Lens Subregions

Except for ENO1, which actually decreases, taxon specific crystallin gene expression tends to increase or remain level from the central epithelia to the fibers (Fig. 2B). ASL1, the predominant crystallin in the chicken lens, is expressed at high levels in all compartments of the 13 dpf chick lens, with a dramatic increase in expression between the equatorial epithelial and peripheral fiber compartments (Fig. 2B) that places it at a unique level if not in a unique pattern (Fig. 2B, Table 2). Both ASL1 and ASL mRNA levels are highly correlated with CRYBB1, CRYBA4, and CRYGN (group 2 in Table 2), as are CRYL1, CRYZL1 and GAPDH, and to some extent ENO1. GSTT1 and to some extent RBP1 are correlated with CRYBB3 and CRYBA1 (group 3), whereas the remaining taxon specific crystallins appear to be independently regulated.

### Comparison of the 30 Most Highly Expressed Genes in Microdissected Lens Subregions

Interestingly, when correlations between expression of crystallin genes in groups 1, 2, and 3 and the other highly expressed lens mRNAs are examined, only 3 (MIF, LOC100857858 and LOC100859737) are closely correlated with the genes in group 1, with VIM, EEF1A1 and ISYNA1 showing a lower correlation (Table 3). In contrast, 19 (BASP1, BFSP1, BFSP2, CD24, CRCP, LGSN, MIP, LOC776816, TPT1, YBX3, RPS11, RPL5, RPS20, RPS23, GAPDH, RPL8, RPL39L, RPL4 and RPS6) are closely correlated with all group 2 genes, and seven more (YBX1, PNRC1, RPS8, ENO1, RPS10, RPL38, and RPS2) show a lower correlation. Finally, CRYBB3 and CRYBA1 (group 3) are closely correlated with each other whereas CST3, LDHA, PABPC1, LOC112530942, and ARIH1 are loosely correlated with CRYBB3 but not CRYBA1. Expression levels of ACTB and LOC101747587 are not correlated with group 1, 2, or 3 crystallins.

**Table 3.** Coefficients of Correlation of the 30 Most Highly Expressed Genes Across the Lens Regions. Highly Expressed Crystallins Are Shown at the Top of the Table. Correlation Coefficients Are Color Coded With *red* > 0.95; *pink* > 0.9; and *blue* < 0.5. Highly Expressed Genes Are Grouped and Colored as in Table 1


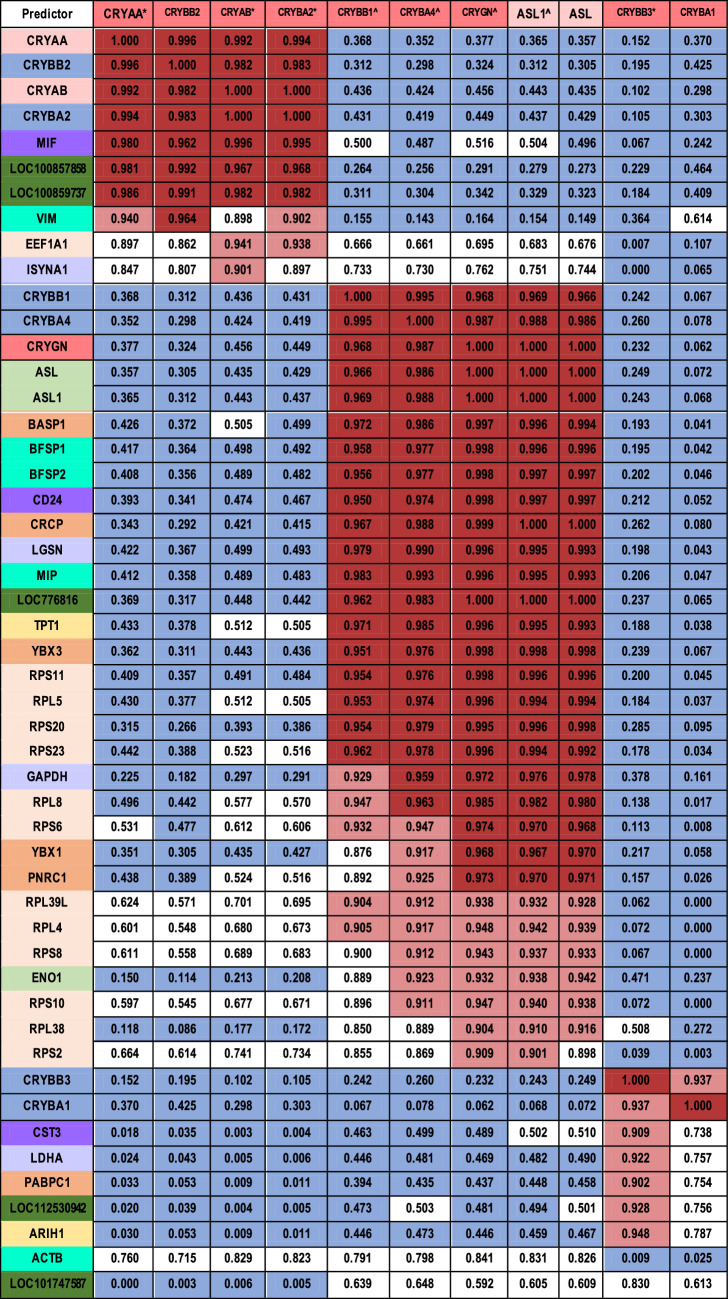

### Transcription Factors Represented in Promoter Regions of Group 1, 2, and 3 Genes

The mRNA levels of four highly expressed transcription, developmental, and RNA processing factors, YBX1, PNRC1, BASP1, YBX3, and the membrane bound receptor CRCP are highly correlated with group 2 of the lens crystallins (Table 3) and are coordinately expressed along with KLF10, NR2C2 and to a lesser extent FOXE3 (Table 4), suggesting that these factors might also have a role in controlling the expression of the genes in group 2. PABPC1, MAF, MAFF, and to a lesser extent MAF1 and Pax6 are also coordinately expressed as a group (*R*^2^ > 0.91 and 0.87 and 0.81 for the main group and MAF1 and PAX6, respectively; Table 4). Other transcription factors known to be active in lens gene expression do not appear to be coordinately expressed with the highly expressed group of transcription factors.

**Table 4.** Correlation Coefficients of Known Lens Transcription Factors. Highly Expressed Transcription Factors Are Shown at the Top of the Table. Correlation Coefficients Are Color Coded With *dark tan* > 0.95; *light tan* > 0.9; and *blue* < 0.5


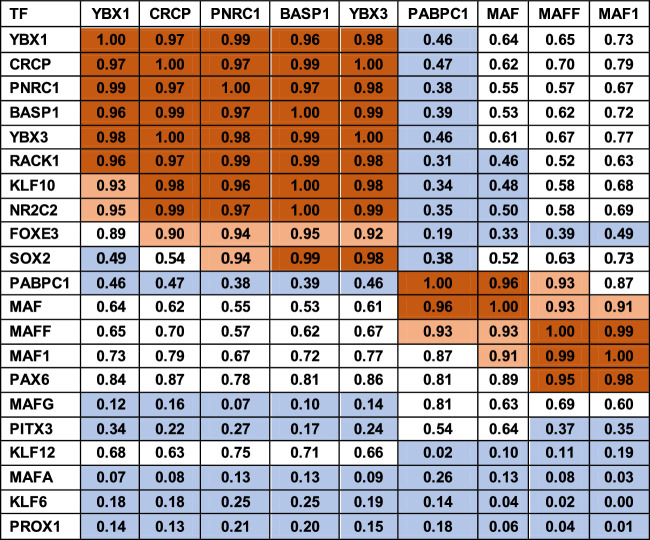

Although binding sites for these factors are not present in the 5’ regions of all the group 2 genes (except for MAF, the binding site for which is present in all members of all 3 groups), binding sites for a number of transcription factor families are found in the 5ʹ 1kb flanking regions of all members of group 1 (Supplementary Table S1A), group 2 (Supplementary Table S1B), and group 3 (Supplementary Table S1C) genes. There are 12 Genomatix matrices unique to group 1 crystallin promoters, six unique to group 2, and 41 unique to group 3 (Supplementary Fig. S3). The Genomatix matrices common to groups 1, 2, and 3 crystallins are shown in Tables 5^6^–7, respectively. Of the 51 Genomatix matrices (representing binding sites recognized by 331 transcription factors) common to group 1 promoter regions, 20 are common to all three crystallin groups, 2 are also found in all group 2 but not 3 gene promoter regions, and 17 are found in all group 3 but not 2 crystallin promoter regions. Of the 331 transcription factors that bind to the matrix sequences in group 1 only 13 are highly correlated with expression levels of the group 1 genes themselves, including four (HSF5, PPARG, SMAD9, and HOXA10), for which the binding site matrices are unique to group 1 (Table 5). Similarly, of the 37 Genomatix matrices (recognized by 271 transcription factors) common to group 2 genes, two are common with group 1 but not group 3, and nine are common with group 3 but not group 1. Of the transcription factors binding matrices common to group 2, 77 are highly correlated with expression of the group 2 genes themselves, of which four (CUX1, NR3C1, NR3C2, and PLAG1) bind to matrices unique to group 2 genes (Table 6). Finally, of the 87 Genomatix matrices (recognized by 528 transcription factors) common to group 3 genes, 17 are common with group 1 but not group 2, and nine are common with group 2 but not group 1. Of the transcription factors binding matrices common to group 3, only 11 are highly correlated with expression of the group 3 genes themselves, of which 4 (NKX6-3, POU4F1, ISL1 and LOC107049603) bind to a matrix unique to group 3 genes (Table 7), although JUN binds to two matrices, one of which is unique to group 3.

**Table 5.** Coefficients of Determination of Transcription Factors Present in All group 1 Genes and *R*^2^ > 0.9 Correlation Coefficients Are Color Coded: *light orange*: 0.9–0.95 and *dark orange*: >0.95 and Unique Matrix Distribution


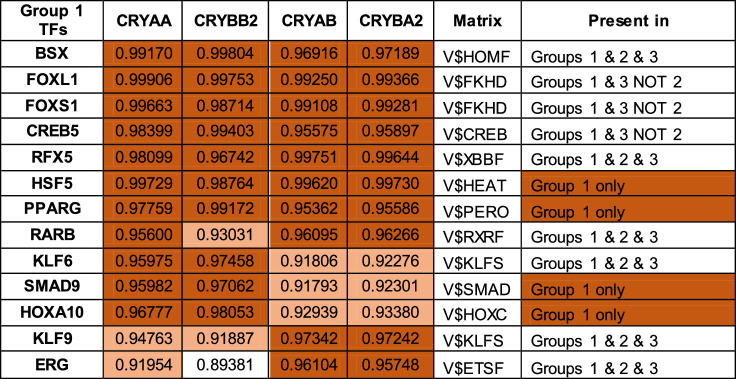

**Table 6.** Coefficients of Determination of Transcription Factors Present in All Group 2 Genes and *R*^2^ > 0.9. Correlation Coefficients Are Color Coded: *light orange*: 0.9–0.95 and *dark orange*: >0.95 and Unique Matrix Distribution


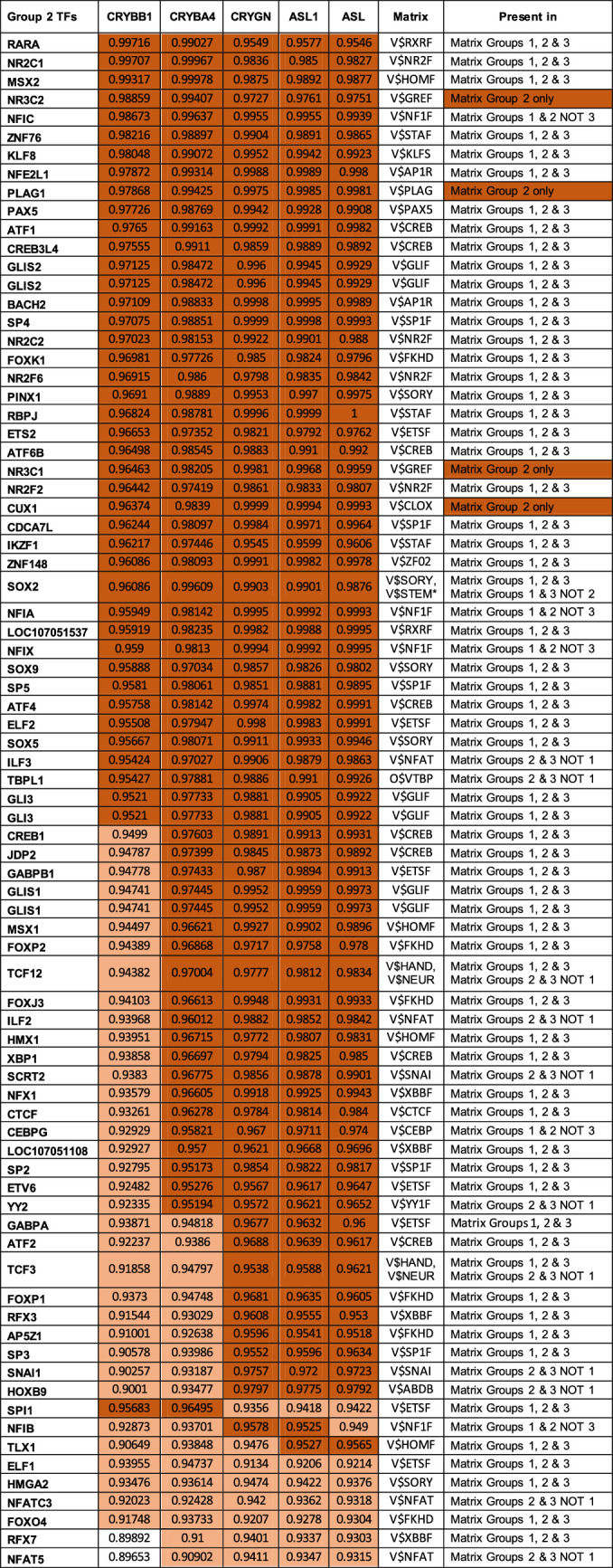

**Table 7.** Coefficients of Determination of Transcription Factors Present in All Group 3 Genes and *R*^2^ > 0.9. Correlation Coefficients Are Color Coded: *light orange*: 0.9–0.95 and *dark orange*: >0.95 and Unique Matrix Distribution


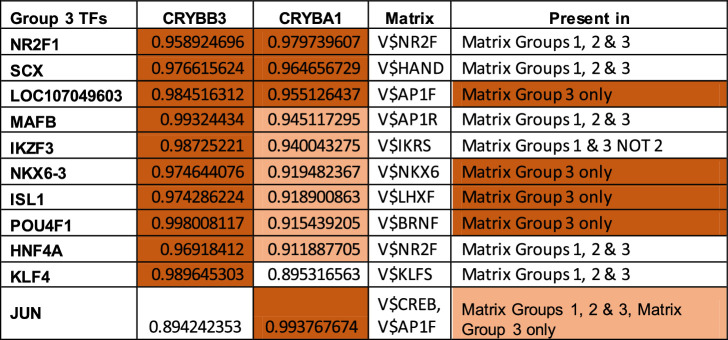

Another way in which to estimate the probability of a transcription factor to be part of the regulatory mechanism for coordinate control of the three groups of genes is by combining the P value of the matrix to which it binds with the average *R*^2^ value of that matrix for all crystallin genes in the group. This is shown for transcription factors with *P* < 0.05 and *R*^2^ > 0.9 in Supplementary Figure S4 and for the larger group of transcription factors with binding sites in all members of each group regardless of *R*^2^ in Supplementary Tables S2A–C. Although there is substantial overlap in the transcription factors identified by these two approaches, there are also some significant differences and reordering of the candidate transcription factors. For example, of the transcription factors predicted to bind to genes of a single group, the only one included in Supplementary Figure S3 is PLAG1 (group 2), although a number of others almost made the cutoff (Supplementary Table S3). However, there is a reasonable overlap between the two approaches with eight group 1–specific transcription factors in common (of 13 and nine in Table 5 and Supplementary Table S2A, respectively), six group 2–specific transcription factors (of 15 and 58 in Table 6 and Supplementary Table S2B, respectively), and one group 3–specific transcription factor (of two and four in Table 7 and Supplementary Table S2C, respectively).

## Discussion

Crystallin synthesis is spatially and temporally regulated in the developing chicken lens, with δ-crystallin appearing first in the presumptive lens ectoderm during placode formation, followed by the appearances of α- and β-crystallins.^24^^–^^26^ Although transient nuclear accumulation of Crybb3 mRNA has been shown in early but not late differentiating mouse lens fiber cells,^27^ transfection and transgenic mouse experiments have indicated that transcriptional control plays a major role in regulation of crystallin gene expression in the lens,^28^ and coupled proteome-transcriptome analysis has shown a high correlation between crystallin mRNA and protein levels.^29^ Thus it is reasonable to use crystallin mRNA levels as a rough metric of crystallin gene expression, if not actual crystallin protein levels.

There are, however, several difficulties in this approach. First, mRNA levels most closely correspond to the rate of synthesis of their corresponding proteins and not their accumulated protein levels. Second, an intrinsic problem in RNA-Seq or other mRNA frequency data is that the estimated mRNA levels are measured relative to the total mRNA pool of the tissue. For example, a mRNA being expressed at a constant level between the EC, EQ, FP, and FC compartments would appear to decrease because of the massive amounts of ASL1 expression, increasing from 2.5% in central epithelia to 16% in central fibers.

One approach to resolving this problem has been normalizing mRNA levels to the DNA in extracted total nucleic acid samples, providing mRNA levels per cell.^30^ However, this is somewhat problematic because the developing fiber cells are elongating and expanding their volume rapidly and beginning to degrade their nuclei during the transition from cortical to nuclear fibers, making normalization difficult. Finally, there is the theoretical possibility of differential mRNA turnover contributing to variations in RNA-Seq estimates, although this seems unlikely given the high prevalence of ASL1 mRNA and its demonstrated long half-life in the chicken lens.^31^ Because of these considerations, we chose to use an average of 14 mRNAs commonly used as controls in qRT-PCR studies.^21^^,^^22^ Because the NE values for these genes varied from 40 (GAPDH) to 0.00059 (ALB), the values for each control gene were first normalized to their value in the central epithelia and then averaged. Other than HMBS, most of the control genes decrease from the EQ to FP compartments, consistent with the increase in ASL1 expression in FC (Supplementary Fig. S2). An additional complication is the possibility that the translation machinery of the lens cells, including ribosomes, initiation factors, elongation factors, and tRNAs, might become rate limiting during this time of massive protein synthesis, increasing competition, and lowering the amount of protein, perhaps differently for each mRNA. This problem is beyond this work, which is limited to analysis at the mRNA level.

The transition from cuboidal epithelial to elongated fiber cells is a complex process in which multiple factors working together regulate cell division, extracellular matrix and integrin production, synthesis of structural proteins including crystallins, and organelle loss.^32^ However, the mRNA expression data shown here suggest that much of the change in lens crystallin mRNAs, both ubiquitous and taxon-specific, occurs by the time the central epithelia have transitioned into equatorial epithelia, and that accumulation of high levels of groups 1 and 3 crystallin proteins in the fiber cells has already been pre-programmed at the transcriptional level at that point or even earlier in the case of CRYL1, ENO1, and lactate dehydrogenase B. In contrast, group 2 crystallin mRNA levels continue to increase from the EQ to FP compartments. The correlation of expression between Cryba4 and Crybb1 in the mouse lens has been noted previously^27^ and felt to be related to the close physical proximity of the genes in a head-to-head orientation (about 3.3 kb, suggesting that they might share promoter or regulatory elements). However, strong correlation among groups 1, 2 and 3 crystallins suggests some further levels of control in transcriptional regulation among members of the three groups.

In the lens, alpha-crystallin plays a dual role as a major refractive element and a molecular chaperone.^33^^,^^34^ In our data, αA-crystallin is among the most highly expressed ubiquitous crystallins. Inoue et al.^35^ showed that in the chicken, while the relative amount of α-crystallin protein to the total lens crystallins is constant throughout development, the ratio of the αA to αB subclasses increases from the embryonic to adult stages. Conversely, our data show that in the chicken, while the fraction of total crystallin mRNA contributed by the α-crystallins decreases from the epithelia to the fiber compartments, probably because of the extremely high levels of δ1-crystallin mRNA, the ratio of the αA- to αB-crystallin mRNAs is constant, remaining about 4:1 across all different spatial regions of the 13d embryonic chick lens.

The β- and γ-crystallins, major components of the vertebrate eye lens, form the βγ-crystallin superfamily sharing a common structure and evolutionary origin from spore coat proteins. However, avian lenses have generally been considered to lack high levels of gamma crystallin. In 2009, Wilmarth et al.^36^ first detected the minor crystallin γN-crystallin in chicken lenses and identified chicken γS-crystallin as the first member of the γ-crystallin family observed in avian lenses. Our data confirm the presence of γS and γN crystallin mRNAs at low abundance levels. Interestingly CRYGS mRNA levels are only very loosely correlated with group 1, whereas those of CRYGN correlate strongly with group 2. Similarly, members of the β-crystallin family are divided among groups 1, 2, and 3, with CRYBB2 and CRYBBA2 in group 1 along with the α-crystallins; CRYB1 and CRYBA4 in group 2 along with ASL1 and 2 and CRYGN; and CRYBB3 and CRYBA1 in group 3. In a previous study, it has been reported that the βA4-crystallin mRNA is present at 400-fold lower levels than the βB1-crystallin mRNA in the 14-day embryonic chicken lens as assayed by Northern blot hybridization analysis.^37^ Our RNA-Seq experiments confirm the lower expression of chicken CRYBA4 relative to CRYBB1 in all sections of the lens, but the ratios were much lower, about 14-fold in the EC, increasing to around 20-fold in the fibers.

The δ-crystallin mRNAs accumulate rapidly during early embryonic development^2^ and decrease after hatching, disappearing from the lens nucleus by about five months.^31^ This suggests that δ-crystallin in avians replaces γ-crystallin in mammals as the main protein component of the densely packed protein-rich lens nucleus.^38^ The δ-crystallin is arginosuccinate lyase,^39^ with two tandemly linked genes (δ1 and δ2) encoding proteins with 91% sequence identity.^40^^,^^41^ Although both genes are expressed to limited extents in non-lens tissues,^42^^,^^43^ the *ASL1* gene codes for the structural crystallin whereas the *ASL* gene codes for the enzyme, and the δ1-protein is approximately 100 times more prevalent in the embryonic chicken lens than δ2-crystallin, possibly because of an enhancer in its third intron.^42^^–^^45^ The ratio of ASL1/ASL in our study was approximately 7 in the EC, rising to 93.5 in the FC, consistent with the observation by Thomas et al.^46^

Other highly expressed genes fall into several groups, generally representing processes that are required for development and elongation of the lens epithelia to form fiber cells. One such process is intermediary metabolism, including glycolysis and the pentose phosphate shunt, represented by LDHA (ε-crystallin), GAPDH, ENO1, and TKT. Although many of the taxon-specific crystallins are derived from enzymes, it seems likely that these four are expressed for their enzymatic activity because their expression levels tend to decrease as the fiber cells differentiate although, GAPDH, like most of the low-expression enzymes, is relatively constant throughout all compartments (Supplementary Fig. S5). Another group comprises structural proteins required for elongation and structural transformation of the lens cells into fibers, including BFSPs, SPARCL1, and ACTB. Interestingly, mRNA encoding VIM, which is replaced by the beaded filament specific proteins in lens fiber cells,^47^ is still present at high levels in central fiber cells.

Finally, there is a rather large group of genes involved in protein expression, including ribosomal proteins and translation factors, as well as a number of transcription factors, including YBX1, which is expressed at disproportionally high levels in the lens. YBX1 roughly correlates with group 2 crystallins (Table 3) but has no binding sites in the 5ʹ regions of any genes in this group. However, genes in groups 1, 2, and 3 each share binding sites for a largely nonoverlapping set of transcription factors, and the mRNA levels of a subset of these are highly correlated with their associated crystallins across the lens subregions. These transcription factors, alone or in combination, would be logical candidates for effecting the correlated expression of genes in groups 1, 2, and 3, as would those in Supplementary Figure S4. Those transcription factors predicted to bind uniquely to promoter regions of crystallins in their associated group would be particularly strong candidates, although transcriptional control by interaction of multiple factors could complicate this simplistic analysis, as could the effects of binding pulse frequency and width, as well as amplitude on transcriptional activation.^48^ In addition, for practical reasons this analysis is arbitrarily limited to transcription factor binding sites within 1 kb of the transcription start site, whereas binding sites further distant are known to influence transcriptional activity and would be missed unless another site belonging to that same family was present within the 1 kb limit.

The correlations of transcription factors with known regulatory roles are generally consistent with this analysis but demonstrate additional complexity in their actions. Examples of this include Pax6, which is critical for development of the eye field, and along with Sox2 activates a set of genes including ASL1 and thus initiates early lens development.^49^^,^^50^ However, as the β-crystallins are expressed Pax6 inhibits CRYBB1 and CRYGF and has complex effects on ASL1 expression,^51^^–^^53^ and Pax6 expression decreases from the EC through EQ to very low levels in the fiber cells (Supplementary Table S3), consistent with the increase in CRYBB1 expression across these regions, thus raising the question of whether Pax6 might have similar inhibitory activity on any of the other group 2 crystallins. In contrast, Sox2, which is known to act cooperatively in binding the ASL1 promoter,^49^ and to activate CRYGF^53^ and ASL1^52^ is tightly correlated with group 2 crystallin mRNA expression as well as PNRC1, BASP1, and YBX3 (Table 4). This interaction is further complicated by Pax6 activating the Sox2 promoter along with AP2 and Prox1.^54^ Similar studies comparing mRNA levels between lens epithelia and fiber cells are generally consistent with these patterns of ubiquitous crystallin and major transcription factor expression, although most studies track expression in epithelia and fibers, and thus a correlation could not be estimated precisely.^55^^,^^56^ However, of the 13 transcription factors correlated with group 1 genes, only six are present in the data reported by Zhao et al.,^56^ and only two of these (CREB5 and KLF6) show increased expression from epithelia to fibers. Similarly, of the 77 transcription factors correlated with group 2 genes, 63 are described by Zhao et al.,^56^ and of these, 11 (MSX2, NR3C2, NFIC, KLF8, NFE2L1, BACH2, SP4, ATF4, GLIS1, YY2, and NFAT5) show increased expression in fibers relative to epithelia, whereas of the 11 transcription factors correlated with group 3 genes, five are described, and only expression of JUN increases from epithelia to fiber cells (Supplementary Table S3).

Alternatively other mechanisms for transcriptional control, including microRNAs and epigenetic regulation such as DNA or histone methylation, that control groups of genes might also be responsible for the coordinated control.^57^ It is notable in this regard that all four crystallin genes in group 1 and both crystallin genes in group 3 are more highly methylated in fibers, whereas the opposite is true of three of the five crystallin genes in group 2 (Table 2, Supplementary Table S4; more detail in Disatham et al., manuscript submitted). The remaining two genes in group 2, ASL1 and ASL, are taxon-specific crystallins that show no changes in methylation. The actual methylation levels and the region of the gene in which the methylation differences occur are shown in Supplementary Table S4.

In a broader sense, E13 days is relatively early in embryonic development, and it seems possible that various regions of the lens epithelia are being defined during this period. This is supported by examination of the pathways prominent in the EC and EQ regions (Supplementary Table S5). Among others, pathways more prominent in the EC include cell cycle components and control, protein processing, and extracellular membrane and cell surface components and interactions. Conversely, pathways more prominent in the EQ include a number of signaling pathways including transforming growth factor-β and some components of the ECM-receptor pathways prominent in the EC. One weakness of this analysis is that dissection of the various lens regions were guided solely by their geographic position in the lens rather than a functional compartmentalization, and a second is that at least in mice, at E13 the germinative region has not defined itself and lens epithelial cell division occurs throughout the epithelia and is even still occurring in differentiating fiber cells,^58^ so that the pathways in specific parts of the lens epithelia might be better defined at a slightly later developmental stage. To accommodate a more-detailed examination of cellular activities that might be carried out by genes expressed at relatively low levels a complete list of genes and their expression levels across the four lens regions is included as Supplementary Table S3.

In conclusion, our results indicate that the level of each crystallin mRNA is controlled differentially and confirm and extend previously reported gene expression patterns for a variety of lens crystallins. The present study identifies three coordinately regulated groups of highly expressed lens proteins and provides a framework for future studies of chicken lenses characterizing developmental, maturational, and pathologic alterations in expression of chicken crystallins and other highly expressed genes.

## Supplementary Material

Supplement 1

Supplement 2

Supplement 3

Supplement 4

Supplement 5

Supplement 6

Supplement 7

Supplement 8

Supplement 9

Supplement 10

Supplement 11

Supplement 12

Supplement 13

Supplement 14
