# Supplementary material for: Patterns of Crystallin Gene Expression in Differentiation State Specific Regions of the Embryonic Chicken Lens
Source: Invest Ophthalmol Vis Sci. 2022 Apr 12;63(4):8. doi: 10.1167/iovs.63.4.8 (PMC9012887; doi:10.1167/iovs.63.4.8)
Supplement: Supplement 1 [file iovs-63-4-8_s001.pdf]

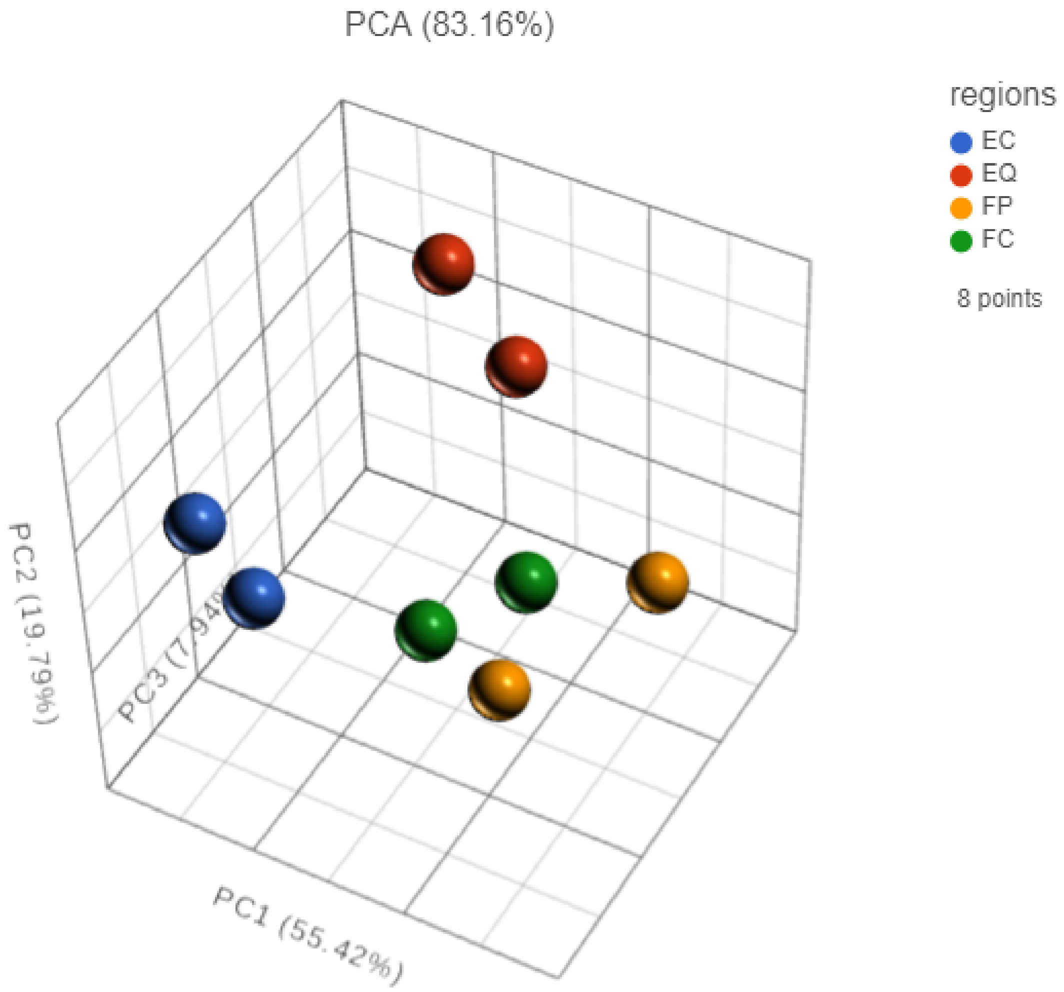

Figure S1. PCA of gene expression in microdissected lens regions. Duplicate samples are color coded: EC (blue), EQ (red), FP (yellow), and FC (green). Duplicates from each region cluster together, and there is little separation of the FP and FC samples, reflecting the minimal changes in mRNA expression between them.
