# Supplementary material for: Patterns of Crystallin Gene Expression in Differentiation State Specific Regions of the Embryonic Chicken Lens
Source: Invest Ophthalmol Vis Sci. 2022 Apr 12;63(4):8. doi: 10.1167/iovs.63.4.8 (PMC9012887; doi:10.1167/iovs.63.4.8)
Supplement: Supplement 2 [file iovs-63-4-8_s002.pdf]

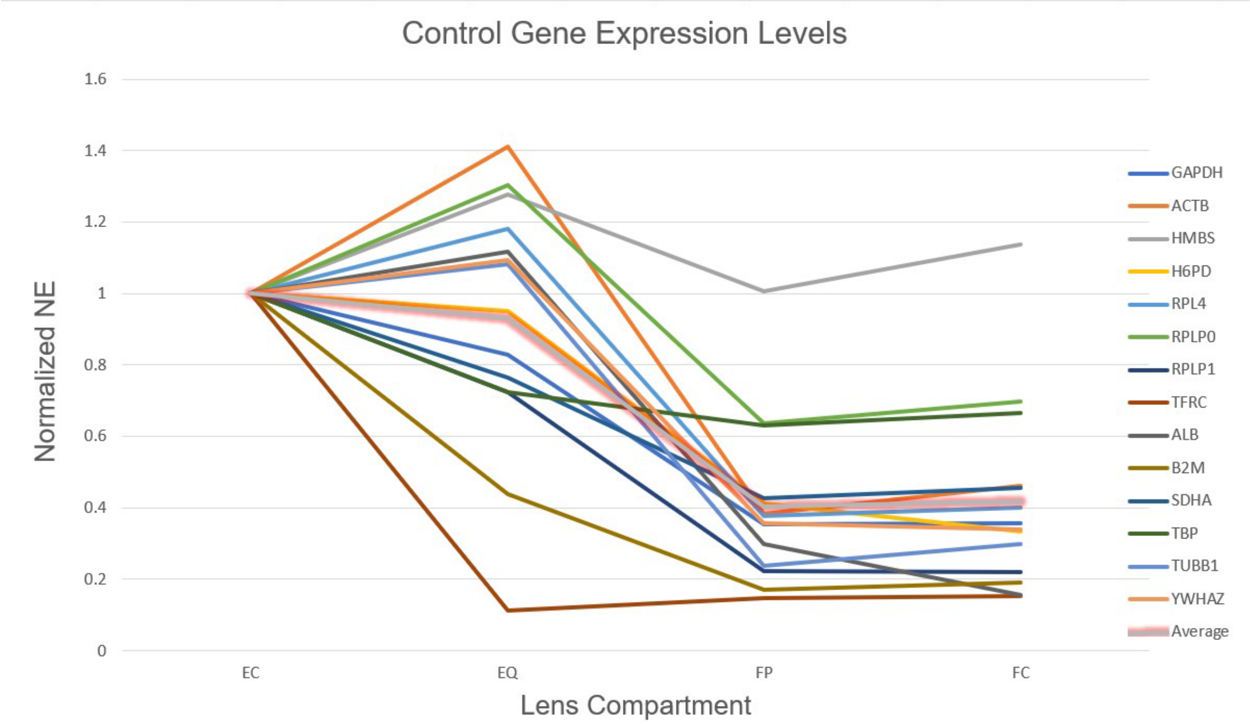

Figure S2. NE values of commonly used control genes normalized to their expression levels in the EC compartment. The control genes included GAPDH (glyceraldehyde-3-phosphate dehydrogenase), ACTB, HMBS, H6PD, RPL4, RPLP0, RPLP1, TFRC, ALB, B2M, SDHA, TBP, TUBB1, and YWHAZ. The average of normalized values of this set of genes, shown above with a halo: 1, 0.92, 0.40, and 0.42 for the EC, EQ, FP, and FC compartments respectively, was used to normalize expression of ubiquitous and taxon-specific crystallins in figure 2 and metabolic enzymes in figure S4, in order to correct for massive levels of ASL1 expression in the fiber compartments.
