# Supplementary material for: Patterns of Crystallin Gene Expression in Differentiation State Specific Regions of the Embryonic Chicken Lens
Source: Invest Ophthalmol Vis Sci. 2022 Apr 12;63(4):8. doi: 10.1167/iovs.63.4.8 (PMC9012887; doi:10.1167/iovs.63.4.8)
Supplement: Supplement 3 [file iovs-63-4-8_s003.pdf]

A.

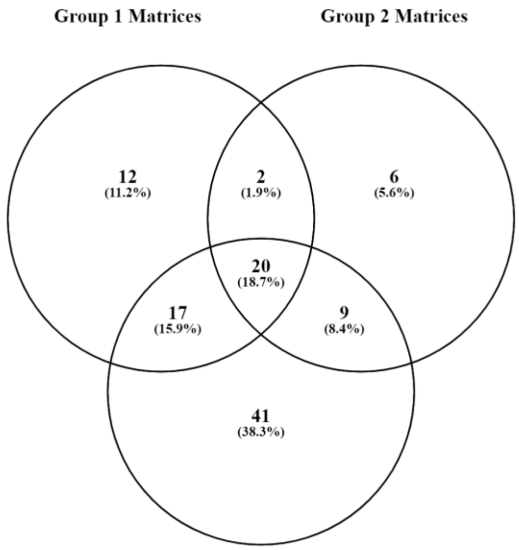

B.

Group 3 Matrices

Matrix Group 1	Matrix Group 2	Matrix Group 3	Matrix Group 3 (cont)	Matrix Groups 1 & 2	Matrix Groups 1 & 3	Matrix Groups 2 & 3	Matrix Groups 1, 2 & 3	Matrix Groups 1 & 2 NOT 3	Matrix Groups 1 & 3 NOT 2	Matrix Groups 2 & 3 NOT 1	Matrix Group 1 only	Matrix Group 2 only	Matrix Group 3 only
V$PAX9	V$AP4R	V$ETSF	V$MYOD	V$ETSF	V$ETSF	V$ETSF	V$ETSF	V$NF1F	V$PAX9	V$EVI1	V$RBP2	V$EREF	V$CHRE
V$RBP2	V$EREF	V$SORY	V$RREB	V$SORY	V$SORY	V$SORY	V$SORY	V$CEBP	V$HEAT	V$GATA	V$DEAF	V$PLAG	V$NKXH
V$DEAF	V$ETSF	V$RXRF	V$PAX5	V$RXRF	V$RXRF	V$RXRF	V$RXRF		V$MYBL	V$ABDB	V$VEZF	V$SRFF	V$WHNF
V$MTF1	V$SORY	V$HOMF	V$RORA	V$HOMF	V$HOMF	V$HOMF	V$HOMF		V$MTF1	O$VTBP	V$INSM	V$P53F	V$STAT
V$ETSF	V$RXRF	V$NR2F	V$IKRS	V$NR2F	V$NR2F	V$NR2F	V$NR2F		V$PAX6	V$AP4R	V$MEF3	V$CLOX	V$GMEB
V$SORY	V$HOMF	V$KLFS	V$RBPF	V$KLFS	V$KLFS	V$KLFS	V$KLFS		V$SREB	V$NFAT	V$SMAD	V$GREF	V$NRF1
V$VEZF	V$NR2F	V$CHRE	V$AHRR	V$CTCF	V$FKHD	V$FKHD	V$FKHD		V$MYOD	V$YY1F	V$MAZF		V$PCBE
V$RXRF	V$SNAI	V$FKHD	V$GCMF	V$FKHD	V$PAX9	V$CREB	V$CREB		V$RREB	V$SNAI	V$LTSM		V$HOXF
V$SREB	V$KLFS	V$PAX9	V$CIZF	V$STAF	V$CREB	V$XBBF	V$XBBF		V$RORA	V$NEUR	V$HOXC		V$HNFP
V$HOMF	V$CTCF	V$NKXH	V$STEM	V$NF1F	V$XBBF	V$OCT1	V$OCT1		V$IKRS		V$PRDF		V$ZF04
V$NR2F	V$FKHD	V$CREB	V$SNAI	V$CREB	V$HEAT	V$HAND	V$HAND		V$STEM		V$CAAT		V$ZF5F
V$INSM	V$STAF	V$WHNF	V$SP1F	V$XBBF	V$OCT1	V$AP1R	V$AP1R		V$HIFF		V$PERO		V$OSRF
V$RREB	V$PLAG	V$XBBF	V$TEAF	V$OCT1	V$MYBL	V$EVI1	V$E2FF		V$AP2F				V$LEFF
V$KLFS	V$SRFF	V$STAT	V$CDXF	V$HAND	V$HAND	V$GATA	V$GLIF		V$EBOX				V$HNF1
V$FKHD	V$NF1F	V$GMEB	V$BRAC	V$AP1R	V$AP1R	V$ABDB	V$PAX5		V$EGRF				V$LHXF
V$AP2F	V$CREB	V$HEAT	V$HIFF	V$ZF02	V$MTF1	O$VTBP	V$GCMF		V$ZICF				V$BRNF
V$MEF3	V$XBBF	V$OCT1	V$AP2F	V$SP1F	V$PAX6	V$AP4R	V$SP1F		V$NFKB				V$PARF
V$CREB	V$OCT1	V$MYBL	V$EBOX	V$CEBP	V$SREB	V$NFAT	V$ZF02						V$NKX1
V$CTCF	V$HAND	V$NRF1	V$FEZF	V$GCMF	V$E2FF	V$YY1F	V$CTCF						V$PAXH
V$XBBF	V$AP1R	V$HAND	V$PDX1	V$PAX5	V$GLIF	V$E2FF	V$STAF						V$HBOX
V$HEAT	V$EVI1	V$PCBE	V$ZF02	V$GLIF	V$MYOD	V$GLIF							V$IRFF
V$OCT1	V$GATA	V$AP1R	V$NEUR	V$E2FF	V$RREB	V$PAX5							V$BRN5
V$MYBL	V$NEUR	V$EVI1	V$ZFHX		V$PAX5	V$GCMF							V$CART
V$STAF	V$ZF02	V$HOXF	V$CTCF		V$RORA	V$SNAI							V$DMRT
V$ZICF	V$P53F	V$HNFP	V$EGRF		V$IKRS	V$SP1F							V$RBPF
V$HAND	V$ABDB	V$MTF1	V$PBXC		V$GCMF	V$ZF02							V$AHRR
V$AP1R	O$VTBP	V$GATA	V$PAX3		V$STEM	V$NEUR							V$CIZF
V$PAX6	V$SP1F	V$ZF04	V$NKX6		V$SP1F	V$CTCF							V$TEAF
V$NFKB	V$CEBP	V$PAX6	V$STAF		V$HIFF	V$STAF							V$CDXF
V$NF1F	V$GCMF	V$ZF5F	V$ZICF		V$AP2F								V$BRAC
V$SMAD	V$CLOX	V$ABDB	V$HESF		V$EBOX								V$FEZF
V$MAZF	V$NFAT	O$VTBP	V$HOXH		V$ZF02								V$PDX1
V$CEBP	V$PAX5	V$OSRF	V$AP1F		V$CTCF								V$ZFHX
V$LTSM	V$YY1F	V$LEFF	V$DLXF		V$EGRF								V$PBXC
V$HOXC	V$GLIF	V$HNF1	V$ESRR		V$STAF								V$PAX3
V$EGRF	V$E2FF	V$LHXF	V$NFKB		V$ZICF								V$NKX6
V$ZF02	V$GREF	V$BRNF			V$NFKB								V$HESF
V$EBOX		V$PARF											V$HOXH
V$HIFF		V$NKX1											V$AP1F
V$PRDF		V$AP4R											V$DLXF
V$E2FF		V$SREB											V$ESRR
V$CAAT		V$PAXH											
V$PERO		V$NFAT											
V$GLIF		V$HBOX											
V$MYOD		V$IRFF											
V$SP1F		V$YY1F											
V$STEM		V$BRN5											
V$PAX5		V$CART											
V$RORA		V$DMRT											
V$IKRS		V$E2FF											
V$GCMF		V$GLIF											

Figure S3. Distribution of Genomatix matrices representing transcription factor binding sites among group 1, 2, and 3 crystallin genes. The matrix names are listed below the Venn diagram.
