# Supplementary material for: Patterns of Crystallin Gene Expression in Differentiation State Specific Regions of the Embryonic Chicken Lens
Source: Invest Ophthalmol Vis Sci. 2022 Apr 12;63(4):8. doi: 10.1167/iovs.63.4.8 (PMC9012887; doi:10.1167/iovs.63.4.8)
Supplement: Supplement 4 [file iovs-63-4-8_s004.pdf]

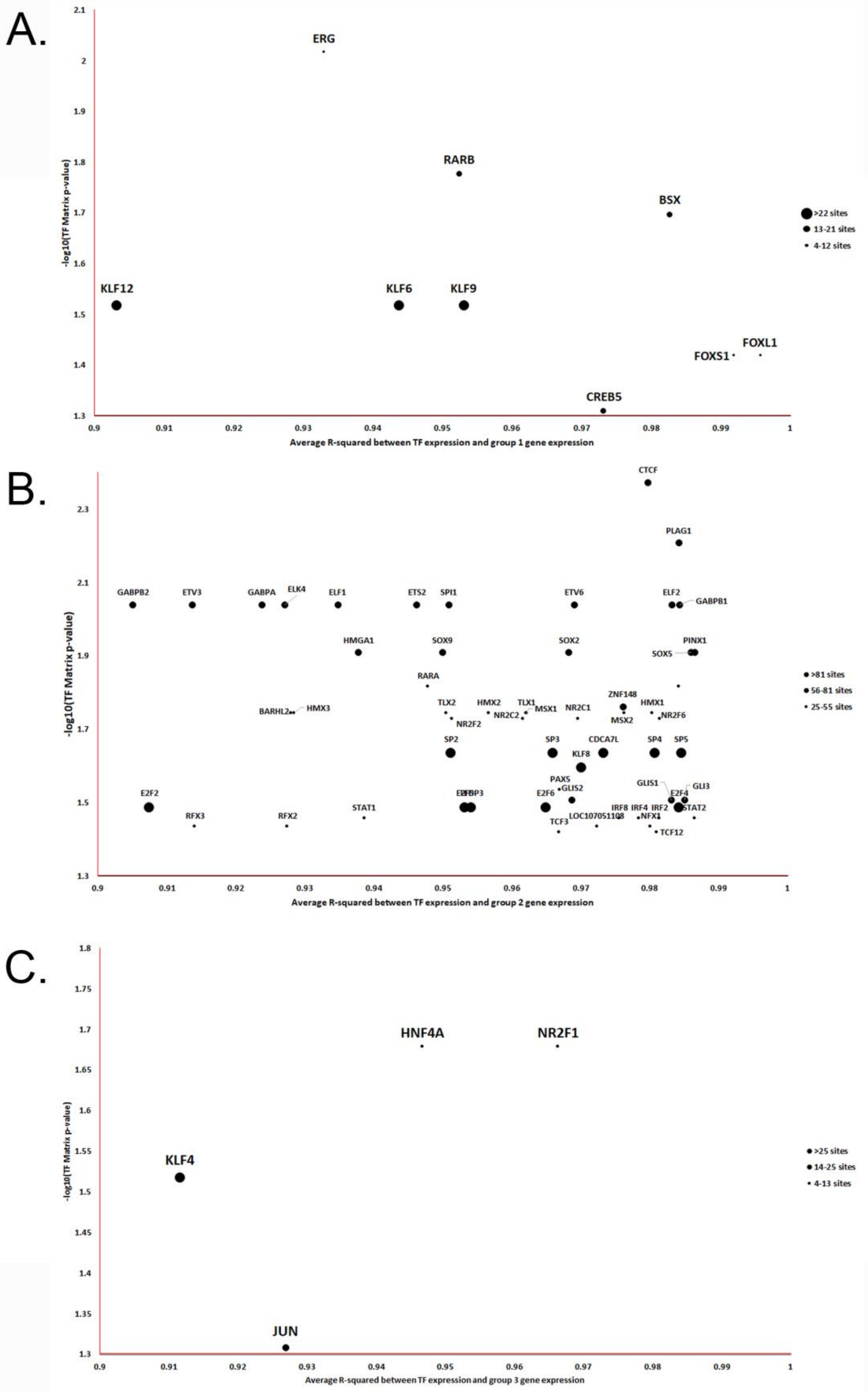
A.
ERG
RARB
BSX
KLF12
KLF6
KLF9
FOXS1
FOXL1
CREB5
>22 sites
13-21 sites
4-12 sites
-log10(TF Matrix p-value)
Average R-squared between TF expression and group 1 gene expression
B.
CTCF
PLAG1
GABPB2
ETV3
GABPA
ELK4
ELF1
ETS2
SPI1
ETV6
ELF2
GABPB1
HMGA1
SOX9
SOX2
SOX5
PINX1
RARA
ZNF148
BARHL2
HMX3
TLX2
HMX2
TLX1
MSX1
NR2C1
HMX1
NR2F6
NR2F2
NR2C2
MSX2
SP2
SP3
CDCA7L
SP4
SP5
KLF8
PAX5
GLIS1
GLI3
E2F2
GLIS2
E2F4
E2F6
IRF8
IRF4
IRF2
STAT2
RFX3
RFX2
STAT1
LOC107051108
NFX1
TCF3
TCF12
>81 sites
56-81 sites
25-55 sites
-log10(TF Matrix p-value)
Average R-squared between TF expression and group 2 gene expression
C.
HNF4A
NR2F1
KLF4
JUN
>25 sites
14-25 sites
4-13 sites
-log10(TF Matrix p-value)
Average R-squared between TF expression and group 3 gene expression

Figure S4. Distribution of transcription factor matrix p values and R2 values for crystallin genes in Group 1 (A), Group 2 (B), and Group 3 (C). Genes with $-\log_{10}$(matrix p values) equal or greater than 1.3 and average R^2 values > 0.9 for Group 1 include FOXL1, FOXS1, BSX, CREB5, KLF9, RARB, KLF6, ERG and KLF12. Genes with $-\log_{10}$(matrix p values) equal or greater than 1.3 and R2 values > 0.9 for Group 2 genes include PINX1, STAT2, SOX5, GLI3, SP5, GABPB1, LOC107051537, E2F4, PLAG1, ELF2, GLIS1, NR2F6, IRF2, TCF12, SP4, HMX1, NFX1, CTCF, IRF4, MSX2, ZNF148, IRF8, CDCA7L, LOC107051108, KLF8, NR2C1, ETV6, GLIS2, SOX2, PAX5, TCF3, SP3, E2F6, TLX1, MSX1, NR2C2, HMX2, TFDP3, E2F5, NR2F2, SP2, SPI1, TLX2, SOX9, RARA, ETS2, STAT1, HMGA1, ELF1, HMX3, BARHL2, RFX2, ELK4, GABPA, RFX3, ETV3, E2F2 and GABPB2 PINX1, STAT2, SOX5, GLI3, SP5, GABPB1, LOC107051537, E2F4, PLAG1, ELF2, GLIS1, NR2F6, IRF2, TCF12, SP4, HMX1, NFX1, CTCF, IRF4, MSX2, ZNF148, IRF8, CDCA7L, LOC107051108, KLF8, NR2C1, ETV6, GLIS2, SOX2, PAX5, TCF3, SP3, E2F6, TLX1, MSX1, NR2C2, HMX2, TFDP3, E2F5, NR2F2, SP2, SPI1, TLX2, SOX9, RARA, ETS2, STAT1, HMGA1, ELF1, HMX3, BARHL2, RFX2, ELK4, GABPA, RFX3, ETV3, E2F2 and GABPB2. Genes with $-\log_{10}$(matrix p values) equal or greater than 1.3 and R2 values > 0.9 for Group 3 genes include NR2F1, HNF4A, KLF4 and JUN.
