# Supplementary material for: Patterns of Crystallin Gene Expression in Differentiation State Specific Regions of the Embryonic Chicken Lens
Source: Invest Ophthalmol Vis Sci. 2022 Apr 12;63(4):8. doi: 10.1167/iovs.63.4.8 (PMC9012887; doi:10.1167/iovs.63.4.8)
Supplement: Supplement 5 [file iovs-63-4-8_s005.pdf]

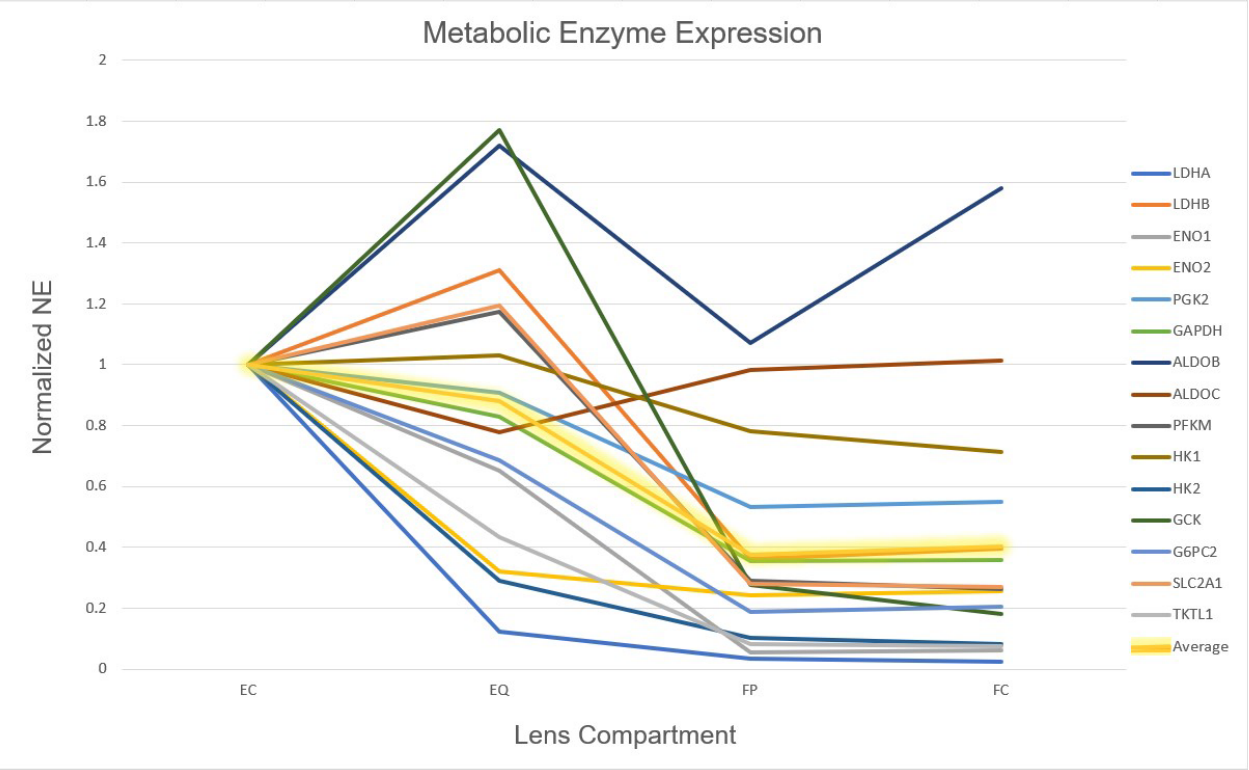

Figure S5. Expression levels of glycolytic and pentose phosphate pathway enzymes. Averages of each group are shown with a halo. The average for these pathways included LDHA, LDHB, ENO1, ENO2, PGK2, GAPDH, ALDOB, ALDOC, PFKM, HK1, HK2, GCK, G6PC2, SLC2A1 and TKTL1. Levels of LDHB and ENO1, both of which are also considered taxon specific crystallins, are shown for comparison. Values are corrected as described for figure 2.
